# Local Energy Decomposition Analysis of London Dispersion Effects: From Simple Model Dimers to Complex Biomolecular Assemblies

**DOI:** 10.1021/acs.accounts.4c00085

**Published:** 2024-04-11

**Authors:** Giovanni Bistoni, Ahmet Altun, Zikuan Wang, Frank Neese

**Affiliations:** † Department of Chemistry, Biology and Biotechnology, University of Perugia Via Elce di Sotto, 8, 06123 Perugia, Italy; ‡ Max-Planck-Institut für Kohlenforschung, Kaiser-Wilhelm Platz 1, 45470 Mülheim an der Ruhr, Germany

## Abstract

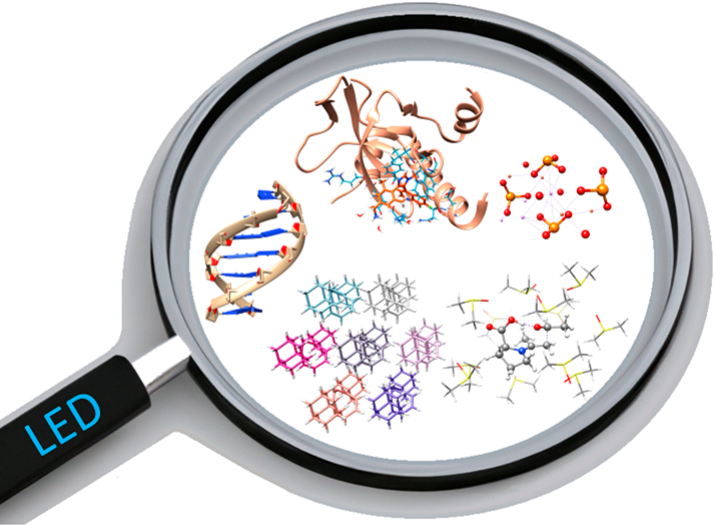

London dispersion (LD) forces are ubiquitous in chemistry, playing a pivotal role in a wide range of chemical processes. For example, they influence the structure of molecular crystals, the selectivity of organocatalytic transformations, and the formation of biomolecular assemblies. Harnessing these forces for chemical applications requires consistent quantification of the LD energy across a broad and diverse spectrum of chemical scenarios. Despite the great progress made in recent years in the development of experimental strategies for LD quantification, quantum chemical methods remain one of the most useful tools in the hand of chemists for the study of these weak interactions. Unfortunately, the accurate quantification of LD effects in complex systems poses many challenges for electronic structure theories. One of the problems stems from the fact that LD forces originate from long-range electronic dynamic correlation, and hence, their rigorous description requires the use of complex, highly correlated wave function-based methods. These methods typically feature a steep scaling with the system size, limiting their applicability to small model systems. Another core challenge lies in disentangling short-range from long-range dynamic correlation, which from a rigorous quantum mechanical perspective is not possible.

In this Account, we describe our research endeavors in the development of broadly applicable computational methods for LD quantification in molecular chemistry as well as challenging applications of these schemes in various domains of chemical research. Our strategy lies in the use of local correlation theories to reduce the computational cost associated with complex electronic structure methods while providing at the same time a simple means of decomposition of dynamic correlation into its long-range and short-range components. In particular, the local energy decomposition (LED) scheme at the domain-based local pair natural orbital coupled cluster (DLPNO-CCSD(T)) level has emerged as a powerful tool in our research, offering a clear-cut quantitative definition of the LD energy that remains valid across a plethora of different chemical scenarios. Typical applications of this scheme are examined, encompassing protein–ligand interactions and reactivity studies involving many fragments and complex electronic structures. In addition, our research also involves the development of novel cost-effective methodologies, which exploit the LED definition of the LD energy, for LD energy quantification that are, in principle, applicable to systems with thousands of atoms. The Hartree–Fock plus London Dispersion (HFLD) scheme, correcting the HF interaction energy using an approximate CCSD(T)-based LD energy, is a useful, parameter-free electronic structure method for the study of LD effects in systems with hundreds of molecular fragments. However, the usefulness of the LED scheme reaches beyond providing an interpretation of the calculated DLPNO-CCSD(T) or DLPNO-MP2 interaction energies. For example, the dispersion energies obtained from the LED can be fruitfully used in order to parametrize semiempirical dispersion models. We will demonstrate this in the context of an emerging semiempirical method, namely, the Natural Orbital Tied Constructed Hamiltonian (NOTCH) method. NOTCH incorporates LED-derived LD energies and shows promising accuracy at a minimum amount of empiricism. Thus, it holds substantial promise for large and complex systems.

## Key References

- SchneiderW. B.; BistoniG.; SpartaM.; SaitowM.; RiplingerC.; AuerA. A.; NeeseF.Decomposition of Intermolecular Interaction Energies within the Local Pair Natural Orbital Coupled Cluster Framework. J. Chem. Theory Comput.2016, 12, 4778–479227564403 10.1021/acs.jctc.6b00523.^1^*In this work, we introduced the Local Energy Decomposition (LED) scheme, describing the underlying theory and its implementation in ORCA*.
- AltunA.; SaitowM.; NeeseF.; BistoniG.Local Energy Decomposition of Open-Shell Molecular Systems in the Domain-Based Local Pair Natural Orbital Coupled Cluster Framework. J. Chem. Theory Comput.2019, 15, 1616–163230702888 10.1021/acs.jctc.8b01145PMC6728066.^2^*In this work, we described the extension of the LED scheme to open shell systems and examined spin effects on the magnitude of the London dispersion (LD) energy on various molecular systems*.
- AltunA.; NeeseF.; BistoniG.HFLD: A Nonempirical London Dispersion-Corrected Hartree-Fock Method for the Quantification and Analysis of Noncovalent Interaction Energies of Large Molecular Systems. J. Chem. Theory Comput.201915, 5894–590731538779 10.1021/acs.jctc.9b00425.^3^*In this work, we introduced the Hartree–Fock plus London Dispersion (HFLD) method for the quantification and analysis of noncovalent interactions in large and complex systems*.
- WangZ.; NeeseF.Development of NOTCH, an All-Electron, beyond-NDDO Semiempirical Method: Application to Diatomic Molecules. J. Chem. Phys.2023, 158, 18410237154284 10.1063/5.0141686.^4^*This work describes the theory and implementation of the NOTCH semiempirical method*.

## Introduction

London dispersion (LD) plays a ubiquitous role in chemistry, exerting influence over the formation of biomolecular aggregates, the selectivity and the rate of chemical reactions, and the structure and properties of energy materials.^5−9^ Owing to its wide-ranging applications in chemical research, the precise quantification of the LD energy has become an increasingly important task in recent years. The ability to evaluate the strength of the attraction between the ligand and the residues in a protein–ligand complex or between different functional groups in a transition state is crucial for advancing the development of new molecules and materials with tailored properties. In this Account, we outline our research endeavors in this context.

From an electronic structure standpoint, the LD energy can be understood as a long-range dynamic electronic correlation. Hence, its rigorous description would require the use of highly correlated wave function-based methods that are capable of describing dynamic correlation accurately. In particular, the coupled cluster method with singles, doubles, and perturbatively included triples correction, CCSD(T),^10^ is often regarded as the “gold standard” of computational chemistry for its high accuracy and broad applicability. Unfortunately, the unfavorable scaling of CCSD(T) with system size has long limited its applicability to small model systems. Our approach to deal with this problem has been rooted in the family of methods stemming from the pioneering contributions of Pulay,^11^ which exploit the short-range nature of dynamic electron correlation to reduce the inherent steep scaling of highly correlated wave function-based techniques. In particular, the domain-based local pair natural orbital CCSD(T) method, DLPNO-CCSD(T),^12^ has demonstrated its robustness, efficiency, and great accuracy for the calculation of electronic energies and other properties in a wide range of chemical applications.^13−16^

However, the ability to compute electronic energies accurately addresses only a part of the problem. Achieving a precise quantification of the LD energy would require differentiation between short-range and long-range dynamic correlation, which is, from a rigorous quantum mechanical point of view, not possible. This renders the LD energy itself a quantum mechanical quantity, with some inherent ambiguity. Nevertheless, the widespread significance of LD in chemical research has stimulated the development of a large number of computational tools for its quantification. Each of these methods relies on specific sets of assumptions, leading to varying numerical values.^17^ Since there is an infinite number of ways to decompose an observable energy into nonobservable components, there is not necessarily a “better” or “worse” in these schemes as long as the energy components behave in a physically correct way (e.g., the dispersion should have *R*^–6^ distance dependence). It is our position that different schemes for isolating the dispersion energy are best judged by the merits these schemes have for understanding and inspiring chemistry. Thus, in our view, the primary objective of this area of chemical research is the development of quantum mechanical methods capable of providing a quantification of the LD energy that (i) is contingent upon the electronic structure of the system and hence responds to alterations in hybridizations, ionizations, or spin effects, (ii) maintains its validity regardless of the strength of the interaction, and (iii) is applicable to all areas of chemical research in which LD plays a fundamental role.

Local correlation methods provide a simple means of discriminating between long-range and short-range correlation by localizing the occupied and virtual orbital space. This approach enables the assignment of molecular orbitals to the fragment (molecules, functional groups, protein residues) in which they are predominantly localized. This, in turn, facilitates the grouping of double excitations that contribute to the correlation energy into distinct families, corresponding to different physical components of the interaction. This strategy is at the basis of the well-established “local energy decomposition” (LED),^1,2,17^ which has been developed with the intent to facilitate the chemical interpretation of the DLPNO-CCSD(T) results. This approach has found widespread applications across various domains of chemical research, some of which are briefly outlined in the following sections. In addition, in this contribution, we will delve into the basic principles and fundamental assumptions behind the LED scheme and discuss how the clear-cut definition of LD energy provided by this method opens up new opportunities for the development of cost-effective electronic structure methods which are potentially applicable to systems with thousands of atoms.

## Local Energy Decomposition

**Figure 1:**
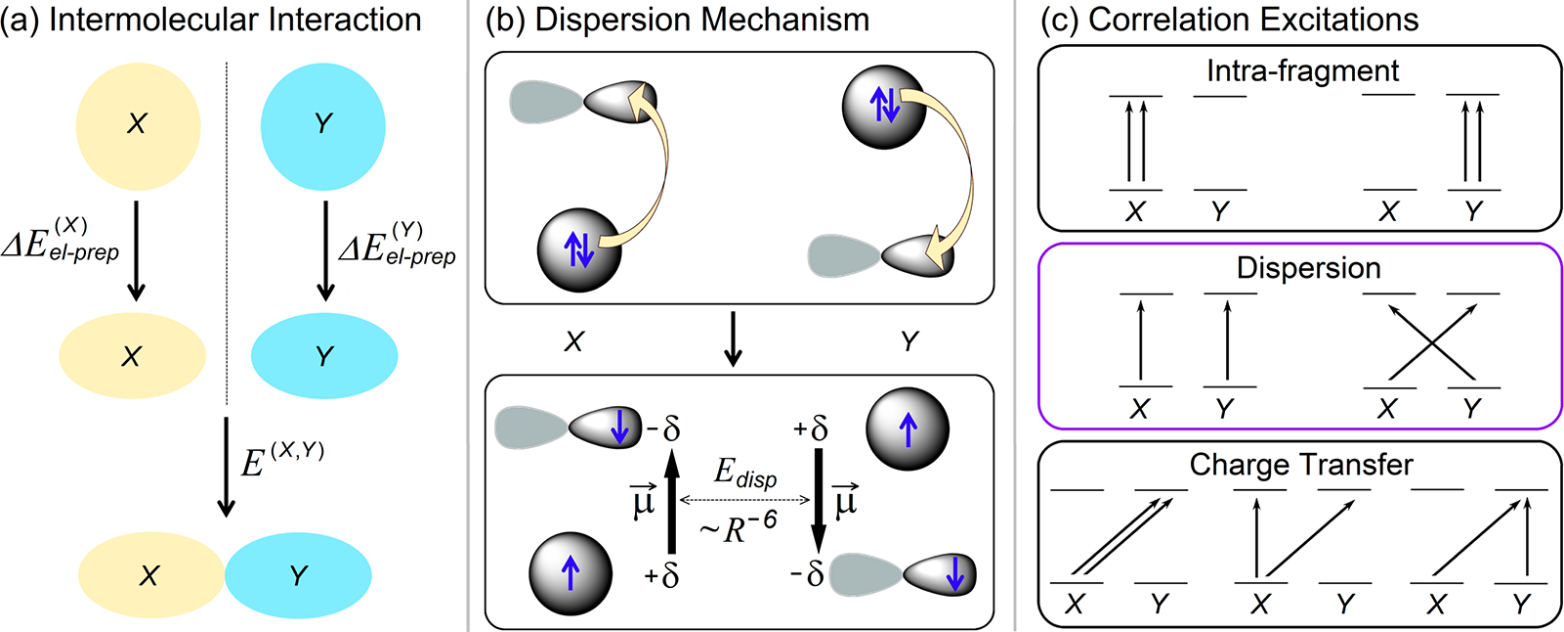
Schematic representation of (a) electronic preparation energies (Δ*E*_el-prep_) and interfragment *E*^(*X,Y*)^ terms associated with the interaction between fragments *X* and *Y*, (b) dispersion forces originating from instantaneous dipole–dipole interactions, and (c) double excitations families constituting the correlation energy from occupied to virtual orbitals (PNOs) in the DLPNO-CCSD(T)/LED method. Dispersion excitations are emphasized in violet.

The first definition of the LD energy within local schemes was initially proposed by Schütz et al. in the context of local MP2.^18^ While this method naturally provided a quantification of the LD energy that responds to the changes in the electronic structure of the system, it was limited in accuracy and applicability due to the underlying MP2 treatment. This limit of MP2-based schemes has stimulated the development of novel strategies for LD energy quantification based on accurate coupled cluster methods.^17^ Among those, the LED scheme is particularly powerful for the great accuracy of the underlying DLPNO-CCSD(T) method as well as for its broad applicability. Within the LED scheme, the DLPNO-CCSD(T) energy (*E*) is partitioned into a series of additive fragment and fragment-pairwise contributions:
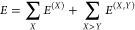
1in which *E*^(*X*)^ denotes the electronic energy of fragment *X* in the system while *E*^(*X,Y*)^ denotes the interaction between fragments *X* and *Y*. This scheme is particularly useful for the decomposition of the relative energies. For example, if we take the electronic energies of the isolated fragments in their electronic ground state (*E*^*X*^) as reference (frozen in their in-adduct geometry), the association energy of the fragments (Δ*E*) can be decomposed as
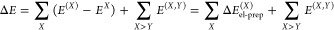
2in which Δ*E*_el-prep_ denotes the “electronic preparation energy”, which is positive by definition and represents the energy required to perturb the wave function of the fragments from their ground state to the one that is optimal for the interaction. It is the dominant repulsive contribution to the association process. A pictorial representation of Δ*E*_el-prep_ and *E*^(*X,Y*)^ is shown in Figure 1a.

A “geometric preparation energy” (also called strain) can also be computed to account for the energy required to distort the fragments from their equilibrium structure to the geometry that is optimal for the interaction. The interfragment terms *E*^(*X,Y*)^ can be positive or negative and can be further decomposed into various physical components, thus yielding additional physical insights:
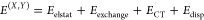
3These include the permanent and induced electrostatic interaction between the fragments (*E*_elstat_), the quantum mechanical exchange (*E*_exchange_), the contribution from correlation excitations of charge transfer character (*E*_CT_), and notably the LD energy (*E*_disp_).

Importantly, the definition of *E*_disp_ and *E*_CT_ exploits the inherent locality of the occupied and virtual orbital space in the DLPNO-CCSD(T) framework to separate long-range and short-range correlation. Specifically, in DLPNO-CCSD(T), the internal orbital space is localized using standard localization procedures, while the virtual space is spanned by a highly compact set of pair natural orbitals (PNOs) that are inherently local and different for each electron pair. To further increase the locality of the virtual space within the LED scheme, these virtual orbitals are subjected to an additional localization procedure. This facilitates the assignment of each occupied and virtual orbital to the fragment where it is predominantly localized. As a result, double excitations contributing to the correlation energy can be categorized into distinct families, aligning with different physical components of the interaction. In this context, the dispersion excitations in *E*_disp_ can be characterized by the presence of one hole and one particle in each fragment (Figure 1b,c), while the charge transfer excitations in *E*_CT_ are identified to those that do not conserve the charge within each fragment.

It is worth mentioning here that in the interest of simplicity, it is often reasonable to combine different LED contributions. For example, in the case of dimers, all the nondispersive terms in eq 3 are sometimes summed to Δ*E*_el-prep_, which leads to a simple decomposition of relative electronic energies into dispersive and nondispersive (e.g., steric/electrostatics) contributions. This simple strategy has found widespread applications in the study of LD effects in various fields of chemical research.^19−22^

Clearly, these features of the LED scheme make it especially useful for the study of noncovalent interactions. However, the energy partitioning outlined above is entirely general, and hence, it has found widespread applications in the most diverse fields of chemical research. In fact, the terms in the decomposition are exactly additive, meaning that their sum yields the total DLPNO-CCSD(T) energy of the system. This remains true for an arbitrary number of fragments regardless of the nature of the system. Hence, the LED scheme provides a theoretical framework in which to discuss LD effects in various domains of chemical research, and the only limit is posed by the accuracy and efficiency of the underlying DLPNO-CCSD(T) treatment.

## Comparison with Alternative Definitions of the LD energy on Standard Benchmark Sets

**Figure 2:**
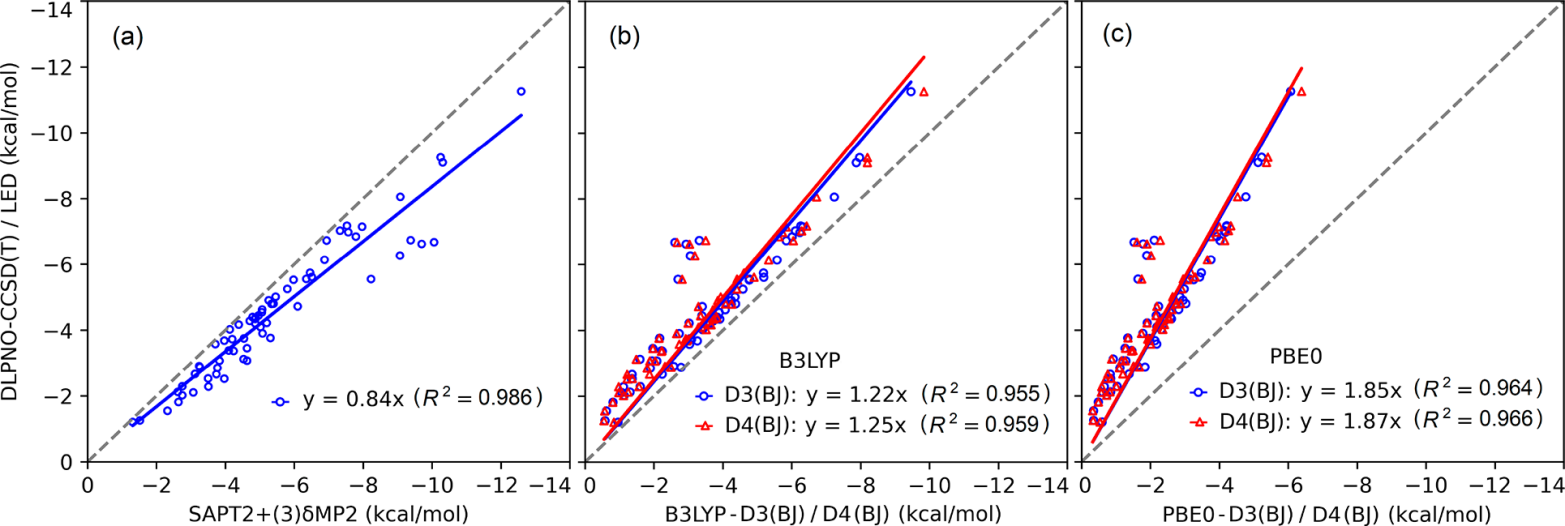
Comparison between the LD energy estimates obtained from LED computed at the DLPNO-CCSD(T)/aug-cc-pVTZ level with that obtained using alternative computational methodologies on the S66 set for noncovalent interactions. (a) LED vs SAPT2+(3)δMP2/aug-cc-pVTZ dispersion (data taken from ref (28)); (b) LED vs D3(BJ) and D4(BJ) dispersion corrections parametrized for B3LYP; (c) LED vs D3(BJ) and D4(BJ) dispersion corrections parametrized for PBE0.

A quantitative one-to-one comparison between the energy components obtained with alternative partitioning schemes is often difficult due to the differing underlying constructions of the theories. However, within the range of applicability of the tested methods, well-defined theories should exhibit similar trends for the LD energy on benchmark sets. Notably, in the weakly interacting regime, symmetry adapted perturbation theory (SAPT) has been used to provide a physically sound definition of the LD energy that has found widespread applications in the study of noncovalent interactions between a pair of interacting monomers.^23,24^ Extending this methodology to encompass, for example, multiple interacting fragments, larger systems, and/or stronger interactions is currently a dynamic area of research.^25,26^ Remarkably, on systems constituting a pair of weakly interacting monomers such as those considered in the S66 benchmark set,^27^ LED estimates for the dispersion energy are numerically consistent with those obtained from SAPT calculations (Figure 2a).

An alternative, widely adopted approach for quantifying the LD energy involves employing “dispersion corrections” such as D3/D4.^29−31^ These or conceptually similar corrections are commonly used in mean-field theories to address the inaccurate dependence of interaction energies on the internuclear distance.^32^ One of the challenges in the development of such methods lies in formulating a definition for the LD energy that responds appropriately to changes in the electronic structure of the system.^29,33,34^ However, for systems with simple electronic structures, such as those in the S66 set, the LED definition of the LD energy is numerically consistent with that obtained by standard dispersion corrections (Figure 2b,c). Importantly, D3/D4 typically underestimates the LD energy with respect to LED and SAPT. This effect is especially noticeable for functionals like PBE, which are already “attractive” and hence are typically associated with smaller dispersion corrections.^35^ The interpretation of these findings is that the “missing” part of the dispersion energy in the D3/D4 correction is already covered by the DFT functional.

In summary, when considering weakly interacting pairs of monomers, the LD energy obtained through the LED scheme aligns numerically with that of the alternative strategies. However, the LED approach stands out as uniquely suitable for addressing challenging chemical applications involving complex systems with multiple interacting fragments and strong interactions. In the next section, we will examine some of these challenging scenarios through illustrative examples from our recent research. Then, we show how the LED definition of the LD energy can serve as a basis for the development of novel and cost-effective electronic structure theories.

## Challenging Applications

**Figure 3:**
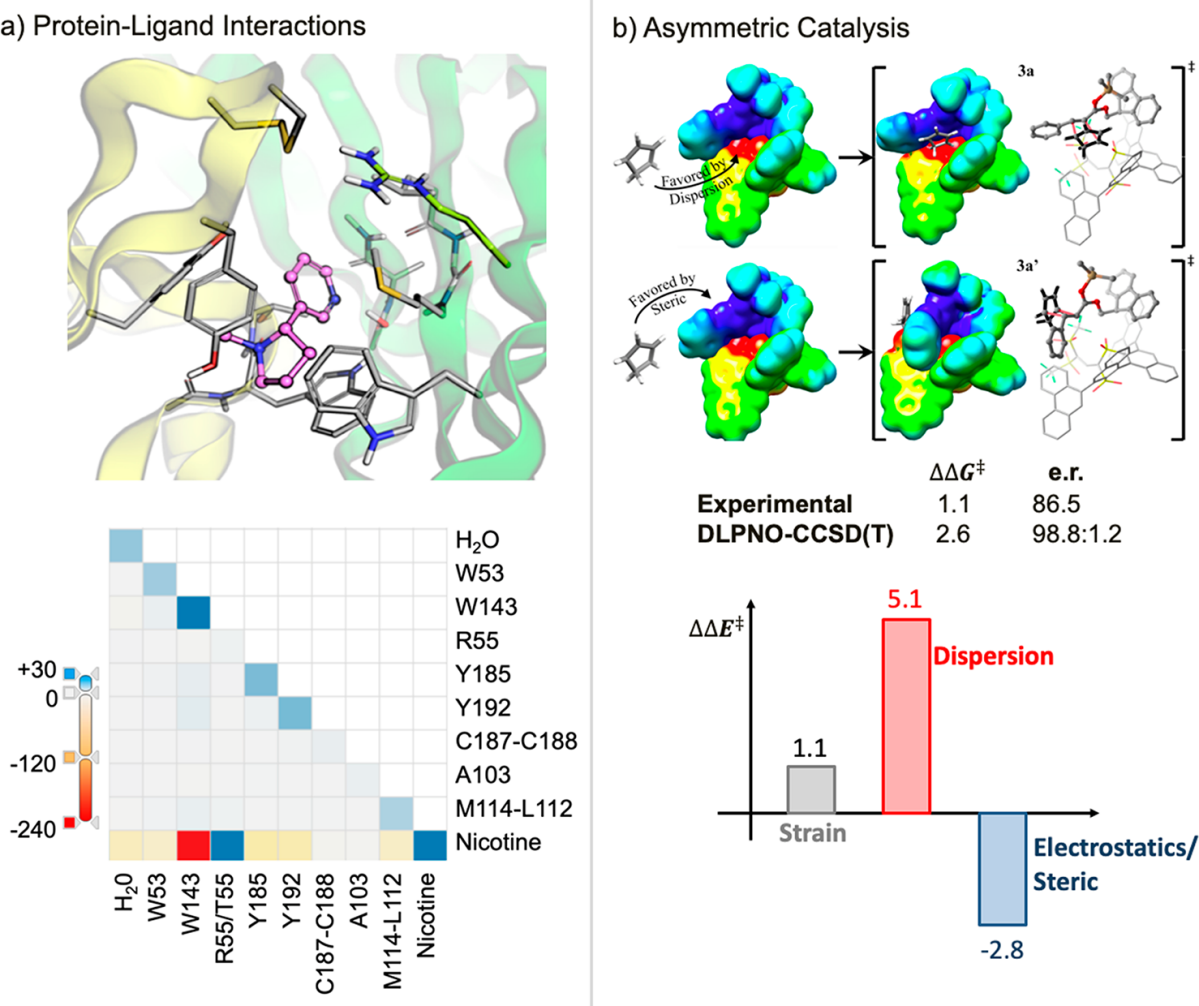
Two illustrative applications of the LED scheme (energies are given in kcal/mol). (a) Protein–ligand interactions for nicotine binding to nAChR protein (upper panel: 3D atomistic model; lower panel: LED interaction map). Adapted from ref (40) under the CC BY 4.0 license. (b) Stereocontrolling transition states for cyclopentadiene attack to a chiral ion pair. Adapted from ref (20) under the CC BY license.

The current research endeavors of our groups encompass the study of complex homogeneous and heterogeneous catalytic transformations, covering a broad range of areas such as bio-, organic, and organometallic chemistry. The LED scheme has become a useful tool in our research, enabling us to discuss diverse chemistries where London dispersion (LD) plays a role within the same interpretative framework.^36−39^Figure 3 showcases two recent illustrative examples taken from our recent research in supramolecular chemistry (Figure 3a) and catalysis (Figure 3b).

One important aspect concerns the study of host–guest interactions, encompassing, for example, the intricate array of noncovalent interactions governing ligand binding to the active site of a protein (Figure 3a, upper panel).^40^ This complex pattern of interactions can be graphically represented by the LED interaction maps (Figure 3a, lower panel). In these maps, the diagonal terms depict the electronic preparation energy (eq 2) on going from the isolated protein and ligand to the protein–ligand adduct and represent the primary repulsive contributions to the interaction. The out-of-diagonal terms delineate fragment-pairwise interactions, representing attractive and repulsive interactions between the ligand and the residues. Extending this scheme to other host–guest interactions and to the study of cooperative and allosteric effects is an important aspect of our current research, with potential applications in the development of highly specific drugs.

In addition to the study of host–guest interactions, the LED scheme has also found widespread applications in catalysis. Importantly, this scheme can be used not only for unveiling LD contributions to the stability of reaction intermediates,^19,21,22^ but also for identifying and quantifying the key covalent and noncovalent interactions responsible for the selectivity of chemical transformations. While a detailed exploration of our extensive work in this field is beyond the scope of this contribution, Figure 3b provides an illustrative example in the context of asymmetric catalysis.^20^

In asymmetric transformations, the experimentally observed enantioselectivity originates from energy differences between activation barriers associated with competing pathways (ΔΔ*E*^⧧^), with each leading to a different enantiomeric product. The LED scheme can be used to quantify the catalyst strain contribution to the selectivity, as well as the role of catalyst–substrate dispersion interactions and electrostatic/steric effects. This can be achieved by decomposing ΔΔ*E*^⧧^ into additive contributions corresponding to each of these distinct physical effects, opening up new avenues for the design of new catalysts with tailored selectivity.

## HFLD

In addition to serving as a useful tool for investigating noncovalent interactions across various research domains, the LED scheme provides a well-defined basis for developing novel electronic structure methods incorporating an effective electronic-structure dependent definition of the LD energy.

For example, in the Hartree–Fock plus London Dispersion (HFLD) scheme,^3^ the HF interaction energy is corrected using an approximated estimate for the coupled cluster LD energy, which is obtained by solving the DLPNO coupled cluster equations while neglecting intrafragment correlation. The resulting LD energy is described by a very small number of correlation excitations (Figure 1c), significantly increasing the efficiency of HFLD compared to that of the full DLPNO-CCSD(T) treatment.

At the core of this approach is the notion that mean-field theories such as HF already capture some essential elements of intermolecular interactions, such as electrostatics and polarization. Electron correlation acts as a correction to these interaction components, while providing at the same time an extra physical contribution that eludes description at the HF level, i.e., the LD energy (note that this notion is already exploited in certain approximated variants of SAPT, such as SAPT0^41^).

**Figure 4:**
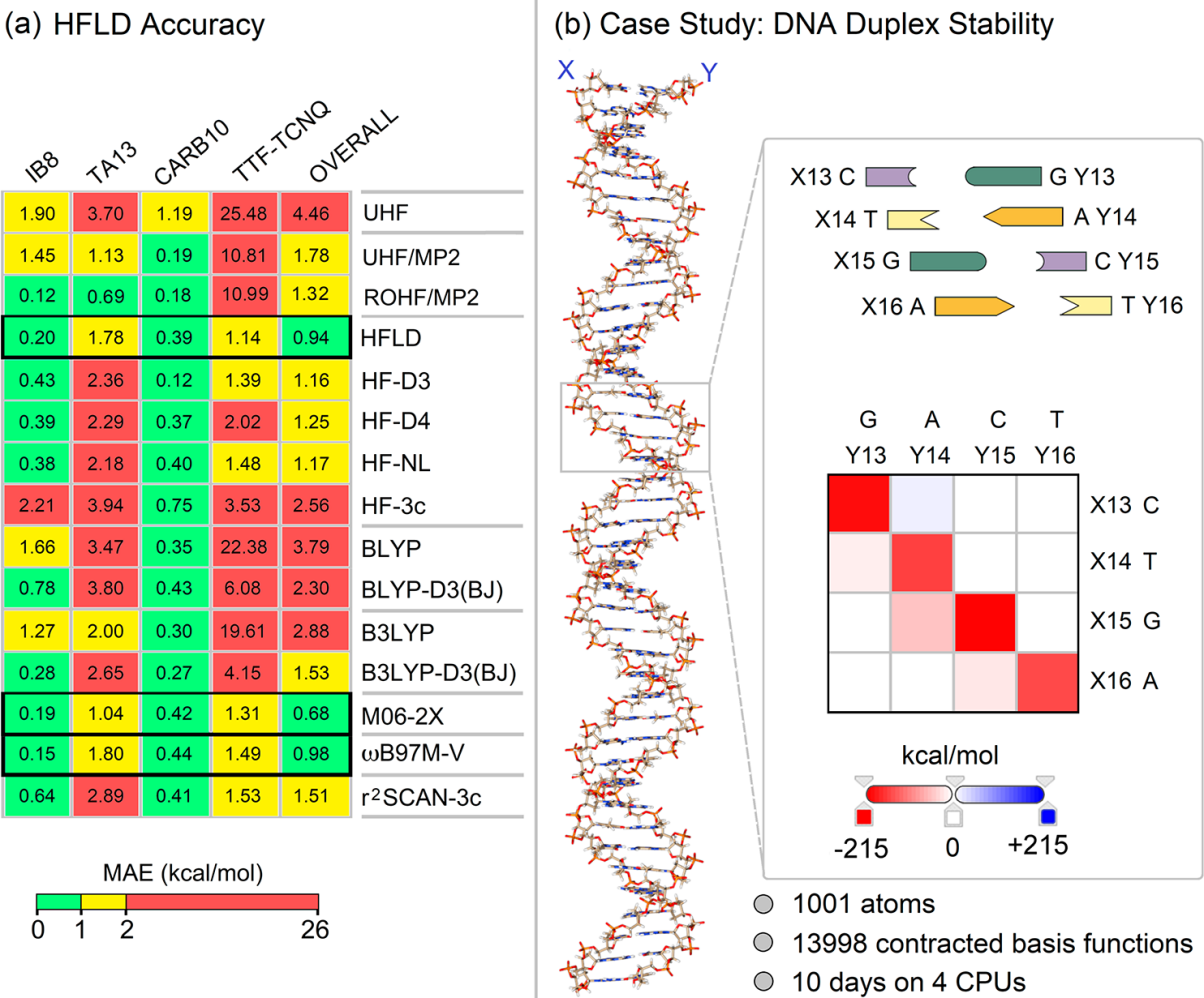
(a) HFLD accuracy in comparison to that of alternative cost-effective electronic structure methods on challenging open-shell benchmark sets. Adapted from ref (42) under the CC BY 4.0 license. (b) A portion of a double stranded human DNA, its schematic ladder, and HFLD/LED interaction energy map. Adapted from ref (38) under the CC BY 3.0 license.

As opposed to some of the alternative approaches for correcting mean-field theories with dispersion terms, such as the semiclassical corrections used in DFT, the LD energy used in HFLD does not contain any empirical parameter. However, neglecting intrafragment correlation introduces two main approximations: (i) an approximation of the estimate of the LD energy, caused by the coupling between the dispersion and intrafragment excitations; (ii) an approximation originating from nondispersive correlation effects.^43^ Remarkably, for noncovalent interactions involving molecules containing main-group elements, these errors tend to cancel each other.^3,42^ As a result, the HFLD method can be considered as one of the most accurate schemes available to date for the study of noncovalent interactions that is applicable to systems of thousands of atoms (Figure 4a).

As an example of the efficiency of this approach, the HFLD scheme has been recently used to study the interaction between two strands of a portion of human DNA (Figure 4b) with 1001 atoms and 13998 basis functions,^38^ showing a computational cost that is comparable to that of the most efficient implementations of mean-field theories.

## NOTCH

In addition to HFLD, another possibility of deriving cost-effective dispersion energy theories is to calculate and fit the DLPNO-CCSD(T)/LED LD energies of some simple systems as a function of molecular geometry and use the fitted functional form to perform calculations on more complex systems. This is different from fitting the total intermolecular interaction energies against high-level data, as is usually done when fitting, e.g., DFT-D parameters.^29−31^ In the latter approach, various deficiencies of the parent method are absorbed into the dispersion correction so that the dispersion correction may not be a very faithful metric of the dispersion energy.

**Figure 5:**
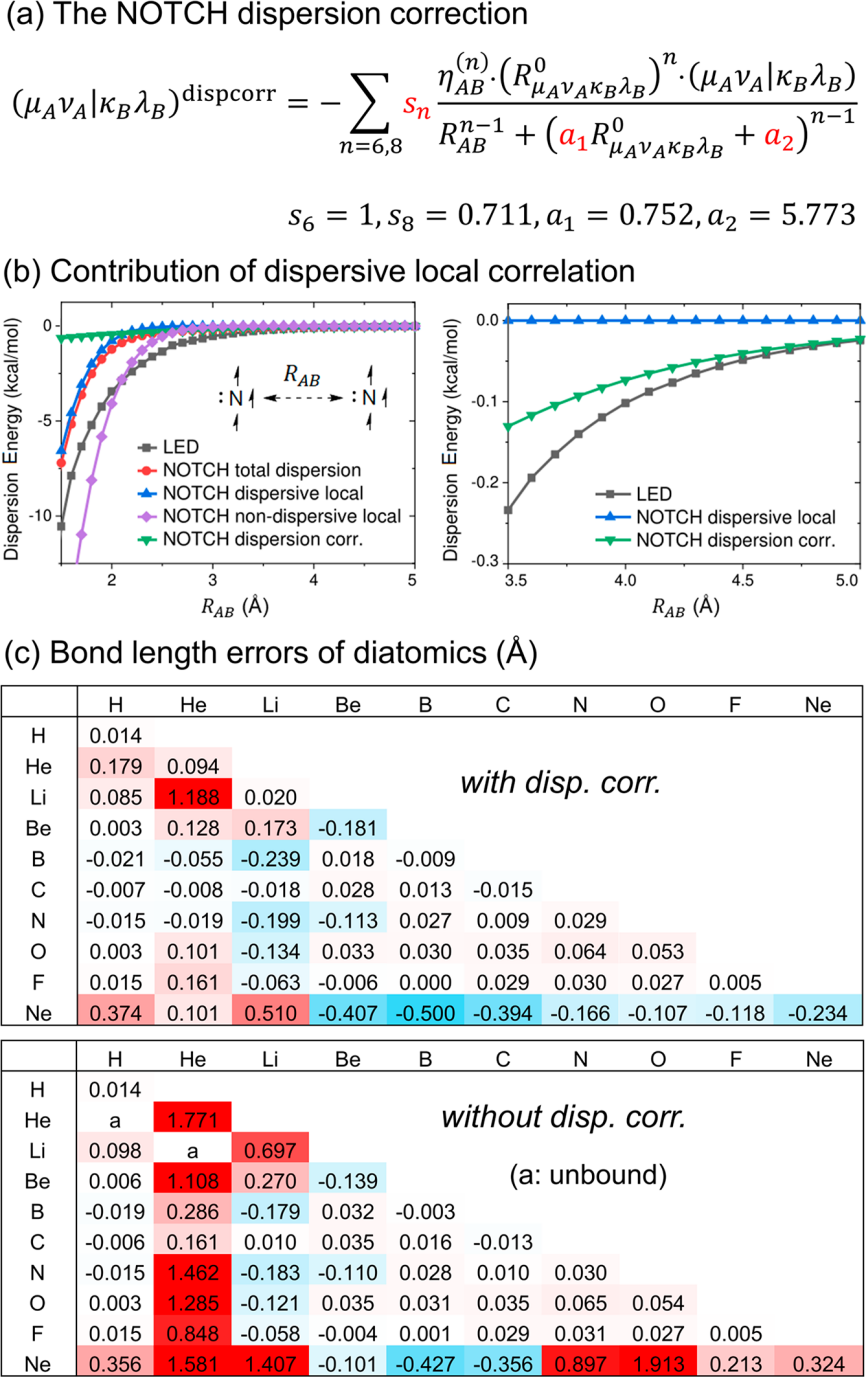
(a) Functional form and fitted parameters of the NOTCH dispersion correction. (b) Comparison of the NOTCH and DLPNO-CCSD(T)/aug-cc-pVTZ LED LD energies of two spin-parallel nitrogen atoms as a function of the interatomic distance. (c) Errors (Å) of NOTCH equilibrium bond lengths of diatomic molecules with respect to CCSD(T) reference values, with and without the dispersion correction. Data taken from ref (4).

This strategy has been used to parametrize the dispersion correction for the Natural Orbital Tied Constructed Hamiltonian (NOTCH) method.^4^ This is a novel semiempirical method, which adopts the form of a minimal basis Hartree–Fock calculation with parametrized integrals and additional corrections.^4^ It is distinguished from existing semiempirical methods by a low level of empiricism as well as the explicit description of many physical effects that are traditionally absorbed into empirical parameters. While a detailed introduction of NOTCH is beyond the scope of this Account, we briefly point out that NOTCH describes dynamic correlation as a local correction plus a dispersion contribution to the two-electron integrals, while left–right correlation and basis set incompleteness are described by other correction terms. Of these, the dispersion correction of the two-electron integrals is the direct equivalent of the dispersion correction in DFT-D-like approaches, with a built-in *R*^–6^ distance dependence (Figure 5a).^4^ However, in NOTCH the correction is performed on the two-electron integrals instead of the energy. Thus, unlike the case of DFT-D, the dispersion in NOTCH does influence the electronic structure of the molecule under investigation. In this respect, it is closer to, for example, the VV10 self-consistent dispersion treatment.^34^

Similar to the case of DFT methods, the division in local dynamic and dispersive correlation contributions is not completely clear-cut. In fact, the NOTCH local dynamic correlation correction also describes some dispersion; even without the dispersion correction, NOTCH still binds rare gas dimers like He_2_, Ne_2_, and HeNe, albeit grossly overestimating their bond lengths (by 1.771, 0.324, and 1.581 Å, respectively; Figure 5c). Therefore, to better compare the NOTCH LD energy with the LED LD energy, one has to separate the dispersive part of the local correlation correction from its nondispersive counterpart and sum the contribution of the former with that of the dispersion correction. In NOTCH, we perform a partition of the local correlation correction under the Löwdin orthogonalized atomic orbital (OAO) basis, similar to what is shown in Figure 1c. Figure 5b illustrates the contributions of the NOTCH dispersion correction, as well as the dispersive and nondispersive parts of the local dynamic correlation, to the noncovalent binding of two spin-parallel nitrogen atoms, compared to the LED LD energies. It is clearly seen that the dispersive local dynamic correlation contributes significantly to (and eventually dominates) the NOTCH LD energy at shorter interatomic distances and is responsible for reproducing the short distance behavior of the LED LD energy. At long distances, however, the dispersion correction dominates the NOTCH LD energy.

Since the NOTCH and LED dispersion energies contain essentially the same physics even at short interatomic distances, we used the LED energies of all 55 neutral atom pairs composed of the elements H–Ne (where the atoms possess parallel spin to prevent bonding) at various interatomic distances as reference data and tuned the 4 empirical parameters in the local dynamic correlation and dispersion corrections to match the NOTCH and LED LD energies. The fitted parameters gave excellent predictions of interatomic distances of all diatomic noncovalent complexes of the elements H–Ne (Figure 5c), including difficult cases such as LiNe, BeNe and HeLi, which many other semiempirical methods overbind strongly.^4^ Note that although we have used exactly the same diatomic molecules in the fitting process, the fit involved only their dispersion energies rather than total energies or equilibrium geometries. Whether the parameters are transferable to multiatomic molecules remains to be seen, but given the success of DFT-D methods,^29−31^ we expect that NOTCH should perform well for general molecules, provided that the coordination number dependence of the atomic C_6_ and C_8_ coefficients is properly treated.

Combined with the success of other terms in the NOTCH method, we were able to achieve comparable or better predictions for the various properties of diatomic molecules than many existing semiempirical methods for diatomic molecules, in particular with much fewer outliers, despite using only 8 empirical parameters for the elements H–Ne, compared to 100–200 empirical parameters in typical semiempirical methods.^4^ Being able to extract the LD energy from the DLPNO-CCSD(T) calculations was evidently a key component of this development. We are currently working on extending the NOTCH method to multiatomic molecules.

## Conclusions

We have been contributing to the understanding and quantification of LD effects across multiple disciplines of chemical research. Our research interests encompass the development of novel computational tools incorporating an electronic-structure dependent definition of the LD energy, as well as challenging applications in supramolecular chemistry, catalysis, and materials science.

Crucial to our research has been the development of Local Energy Decomposition (LED) at the DLPNO-CCSD(T) level. The LED method, by exploiting the “localizability” of dynamic electron correlation, allows for a straightforward separation between short-range and long-range dynamic correlation, thus providing a simple yet powerful definition of the LD energy.

On the S66 benchmark set for noncovalent interactions containing weakly interacting model dimers, comparisons with alternative LD energy definitions, such as that provided by symmetry-adapted perturbation theory (SAPT), have corroborated the consistency and reliability of the LED approach. Importantly, the main strength of the method lies in its applicability across challenging scenarios involving multiple interacting fragments of arbitrary strength. For example, the LED scheme has emerged as a powerful tool for studying host–guest interactions, providing detailed insights into the complex patterns of covalent and noncovalent interactions responsible for protein–ligand binding. Additionally, in catalytic transformations, the LED scheme has found applications in the identification and accurate quantification of the key covalent and noncovalent interactions contributing to intermediate stability and reaction selectivity, opening up new avenues for catalyst design.

Another significant aspect of our research lies in the development of approximated methods that exploit the definition of the LD energy provided by the LED scheme but are potentially applicable to even larger systems with thousands of atoms. Our efforts have led to the development of novel electronic structure methods such as the Hartree–Fock plus London Dispersion (HFLD) and the NOTCH semiempirical method. The HFLD scheme has already found various applications in chemical research and can be regarded as a very accurate tool for the study of LD effects in systems with 500–1000 atoms. While the development of the NOTCH scheme is still in its infancy, its success in describing LD effects and other molecular properties with minimal empiricism underscores its potential as a valuable tool for the study of even larger and more complex systems.
